# Supplementary material for: Educational outcomes of recess in elementary school children: A mixed-methods systematic review
Source: PLoS One. 2023 Nov 22;18(11):e0294340. doi: 10.1371/journal.pone.0294340 (PMC10664954; doi:10.1371/journal.pone.0294340)
Supplement: S1 File — (PDF) [file pone.0294340.s002.pdf]

A systematic review of the effect of school recess on educational outcomes

To enable PROSPERO to focus on COVID-19 submissions, this registration record has undergone basic automated checks for eligibility and is published exactly as submitted. PROSPERO has never provided peer review, and usual checking by the PROSPERO team does not endorse content. Therefore, automatically published records should be treated as any other PROSPERO registration. Further detail is provided here.

Citation

Erin Howie, Laura Cameron. A systematic review of the effect of school recess on educational outcomes. PROSPERO 2021 CRD42021221579 Available from:
https://www.crd.york.ac.uk/prospero/display_record.php?ID=CRD42021221579

Review question

What is the effect of recess on the educational outcomes of elementary school students? Educational outcomes may include academic, cognitive, or behavioral outcomes.

Searches

We will search the following electronic bibliographic databases: ERIC (EBSCO), ProQuest Central, PsycINFO, Teacher Reference Center, MEDLINE Complete (EBSCO), and CINAHL Complete.

The search strategy will include terms related to recess. The search strategy for ERIC (EBSCO) has been supplied as an attachment. The search strategy will be adapted for use with other bibliographic databases.

No language restrictions will be used in the search strategy. Results will be limited by date, with all results published before 2009 excluded. Initial searches will begin in December 2019 and the searches will be re-run preceding the final analysis to retrieve any additional studies for inclusion.

In addition to searching electronic bibliographic databases, a hand search will be executed to retrieve additional studies for inclusion. The hand search will include examining bibliographies of included articles.

Types of study to be included

Primary experimental, longitudinal, or cross-sectional studies of any design (e.g. RCT, quai-experiemental, cross-sectional)

Condition or domain being studied

Child health including academic, cognitive, and behavioral outcomes

Participants/population

Elementary (primary) school students

Intervention(s), exposure(s)

Recess, as defined as a regularly scheduled period in the school day for physical activity and play that is monitored by

trained staff or volunteers

Comparator(s)/control

Not applicable

Context

Elementary (primary) schools in any country, including outdoor play spaces

Main outcome(s)

Educational outcomes defined as academic performance or achievement, cognitive performance, or behavioral outcomes

Measures of effect

Not applicable due to diverse study designs

Additional outcome(s)

None

Measures of effect

Not Applicable

Data extraction (selection and coding)

Two reviewers will independently assess all included articles for data extraction and methodological quality. In case of any disagreement, consensus will be reached during a meeting. If agreement could not be reached, discussion will be held with a third reviewer to decide on the matter. For data extraction, the following data of each included article will be obtained: first author and year of publication, study design, study location, study population (n, age, sex), recess descriptors, outcome construct, outcome measure, main outcome results

Risk of bias (quality) assessment

The GRADE (Grading of Recommendations, Assessment, Development and Evaluation) framework will be used to assess risk of bias for both experimental and observational studies. It will include an assessment of factors that reduce the quality of evidence (eg. study limitations, inconsistency of results, indirectness of evidence, imprecision, and publication bias) and those that increase the quality of evidence (eg. large magnitude of effect, dose-response gradient, effect of plausible residual confounding).

Strategy for data synthesis

It is expected there will be heterogeneity among the included studies., thus key study characteristics and outcomes will be summarized in narrative format as well as evidence tables. Findings will be presetned descriptively, as well as quantitatively by study type and outcome construct.

Analysis of subgroups or subsets

If there are sufficient data, a sub-group analysis or narrative synthesis will be conducted based on for example, country of study or outcome assessment.

Contact details for further information

Erin Howie
ekhowie@uark.edu

Organisational affiliation of the review

University of Arkansas

Review team members and their organisational affiliations

Dr Erin Howie. University of Arkansas

Ms Laura Cameron. University of Arkansas

Collaborators

Ms Tkeyah Mcdaniel. University of Arkansas

Ms Alexis Garner. University of Arkansas

Mr Charles Rodgers. University of Arkansas

Type and method of review

Systematic review

Anticipated or actual start date

06 December 2019

Anticipated completion date

31 May 2021

Funding sources/sponsors

Unfunded

Conflicts of interest

Language

English

Country

United States of America

Stage of review

Review Ongoing

Subject index terms status

Subject indexing assigned by CRD

Subject index terms

Humans; Recreation; Schools

Date of registration in PROSPERO

13 February 2021

Date of first submission

13 January 2021

Stage of review at time of this submission

Stage	Started	Completed
Preliminary searches	Yes	No
Piloting of the study selection process	Yes	Yes
Formal screening of search results against eligibility criteria	Yes	No
Data extraction	No	No
Risk of bias (quality) assessment	No	No
Data analysis	No	No

The record owner confirms that the information they have supplied for this submission is accurate and complete and they understand that deliberate provision of inaccurate information or omission of data may be construed as scientific misconduct.

The record owner confirms that they will update the status of the review when it is completed and will add publication details in due course.

Versions

13 February 2021

13 February 2021
